# Pilot test of an educational intervention to improve self-management of diabetes in persons living with HIV

**DOI:** 10.1186/s40814-019-0495-5

**Published:** 2019-09-07

**Authors:** Julie Zuñiga, Alexandra A. García, Luisa Silva, Jung-Min Park, Yuri Barrera

**Affiliations:** 1 The University of Texas, Austin, USA; 2 The University of Texas at Austin, Dell School of Medicine, Austin, Texas USA

**Keywords:** HIV, Type 2 diabetes, Self-management, Chronic disease management, Health behavior, Nurse, Social worker

## Abstract

People living with a diagnosis of HIV (PLWH) and type 2 diabetes (T2DM) can experience a synergistic negative impact on their vascular and immune systems if their conditions are poorly controlled. The purpose of this study was to adapt a community-based diabetes self-management intervention for people living with HIV and test the feasibility of administering the intervention with PLWH+T2DM who are low-income, predominantly minority, vulnerable population. The intervention was 12 weeks long with 6 h of educational instruction followed by 6 weekly support telephone calls to reinforce training and problem solve. The study used a one-group pretest–posttest design. Participants were a convenience sample of 25 adults diagnosed with HIV + T2DM. Diabetes knowledge, HIV knowledge, and self-management skills were measured. Analyses comprised descriptive statistics and correlations. Participants completed an average of 2.7 of 6 h of instruction and an average of 3 of 6 possible telephone calls. There was a 34% increase in diabetes self-management skills from pretest to posttest, but there were no changes in knowledge about HIV or diabetes. Based on this pilot study, next steps will include a multi-modal educational intervention, with in-person, at-home, and teleconference components. Blood sample collection procedure will be coordinated with study visits to decrease participants’ burden, and the updated diabetes knowledge instrument with a higher reported internal consistency will be used.

## A pilot to improve self-management of diabetes in persons living with HIV

Currently, over 1.2 million Americans are living with HIV [1], and, owing to the efficacy of antiretroviral medications, HIV is now considered to be a chronic condition that can be managed successfully rather than a fatal acute infection [2]. A new challenge to HIV management has arisen; however, depending on age, about 60% of persons living with HIV (PLWH) will be diagnosed with a co-morbid medical condition [3, 4]. Type 2 diabetes (T2DM) is one of the most commonly diagnosed co-morbid conditions affecting the HIV-positive population; approximately 15% of PLWH have a diagnosis of diabetes [5].

PLWH are at greater risk for T2DM than the general population, because HIV infection causes chronic inflammation that persists even after HIV treatment is begun [6]. Sustained systemic inflammation in PLWH is associated with increasing incidence of T2DM [7]. Furthermore, HIV medications cause impaired glucose tolerance, which frequently leads to T2DM [8–10]. Individually, both HIV and T2DM negatively impact the vascular and immune systems, and the synergistic effect of having both diseases leads to still worse health outcomes [10, 11].

## Self-management of diabetes in persons with HIV

Persons diagnosed with both HIV and T2DM (PLWH+T2DM) have difficulty controlling their diabetes while also managing HIV [12]. Self-management of HIV + T2DM can be difficult because of the complexity of both medical regimens, the large amount of information that the management of both conditions requires, the potential for conflicting guidance from multiple health care providers, and treatment side effects and symptoms. Uncontrolled T2DM can lead to a propensity for infections and to long-term complications such as neuropathy, nephropathy, blindness, and amputations caused by vascular damage [13]. Han et al. [14] reported that once started on diabetes medication, PLWH (*n* = 286) were less successful in reducing their glycosylated hemoglobin (A1c), a measure of diabetes control, than were patients without HIV (*n* = 858). Chu et al. [3] found that PLWH+T2DM (*n* = 423) were less likely to achieve treatment goals than were patients with HIV and other co-morbidities.

Interventions to improve self-management of either HIV or T2DM have been successful in helping patients develop self-management skills [15, 16]. Successful self-management of HIV includes many of the same health-promoting behaviors as those used in T2DM self-management, such as taking medications, managing stress, eating healthy foods, and engaging in appropriate physical activity [16]. However, some issues, such as coping with stigma and disclosure of medical status, are more complex for PLWH, given society’s fear of contagion from HIV [16, 17].

In this feasibility trial, the research aim was to explore trial design, acceptability of the outcome measures and to provide data to estimate the parameters required to design a definitive randomized control trial. The primary objectives of the pilot were as follows:

1. To adapt a successful community-based self-management intervention for people with diabetes [15] and
2. To test the feasibility of administering the new, contextually relevant, interactive, group educational self-management intervention for PLWH+T2DM for a low-income, predominantly minority, vulnerable population.

The secondary objectives of the pilot were as follows:

1. To create procedures for data collection and intervention implementation that were acceptable to the participants, as indicated by participation.
2. To investigate the diabetes instruments usefulness in the population with a dual diagnosis of HIV.

## Methods

### Design

We first convened a focus group to gather input on how to tailor the diabetes intervention for PLWH+T2DM. Then, we administered the adapted intervention using a single-group pretest–posttest design. Data were collected from January to September 2016. The research protocol was approved by the University of Texas at Austin Institutional Review Board (study number: 2015-05-0104).

### Focus group

The focus group with PLWH+T2DM (*n* = 6) provided patients’ perspectives on managing both conditions and on components that might make the intervention successful. Participants were recruited from a local HIV clinic where the focus group was held. Prior to beginning the focus group, participants provided signed informed consent. The focus group was conducted by two members of the research team as a structured interview to gain information about topics of interest and preferences for locations, time, and the intervention itself. We asked specific questions about how long the focus group participants would want the intervention to be, including the length of the educational sessions and how many weeks they might be able to commit to attendance. The researchers took extensive field notes, and the session was also audio recorded. At the end of the focus group, participants received a cash incentive and were invited to participate in the intervention once it was revised. Descriptive content analysis approach, focusing first on the frequency of words and content, allowed the researchers to use participants’ feedback to select key topics as well as acceptable logistics for the educational session [18].

### Study sample and setting

The pilot study participants were a convenience sample of 25 adults age ≥ 18 years and diagnosed with HIV + T2DM. Since this is a pilot study, power analysis was not conducted in order to calculate sample size [19]. The researchers aimed for 25 participants because the study had to be completed in 12 months in accordance with funding mechanism guidelines and were further constrained by budget limitations. All participants were able to read, speak, and understand English, and they reported that they were able to access transportation and telephone services. They were recruited in Austin, Texas, from two Federally Qualified Health Centers (FQHCs) for PLWH, a non-profit HIV community service center, a local food bank for PLWH, and through referral by HIV health care providers. Most of the research activities took place at a geographically accessible well-known FQHC HIV clinic that provides care for under- or non-insured PLWH, where most of the participants received care.

### Procedures

Trained registered nurses who were graduate research assistants recruited and enrolled the participants and collected baseline and follow-up data. Baseline data were gathered the week prior to participants’ beginning the intervention. After providing written consent, participants supplied demographic information, responded to survey questions, and provided a blood sample. The survey questions focused on self-management and knowledge of the two conditions. Data collection occurred most often in participants’ homes, but the blood samples were collected at commercial laboratory testing centers. Blood samples were used to assess current disease control—A1c for diabetes and CD4 for immune function. Post-test data were gathered within 3 weeks of the final telephone. Participants who attended all educational sessions and both study visits received $120 in cash.

### Theoretical model

The intervention was informed by the chronic care model [20], which posits that effective self-management support includes assistance with self-management skill building, using the community’s and health care system’s resources and supporting patients’ being informed and activated. The model was created to address the common challenges of chronic illnesses, including difficult medication regimens and lifestyle adjustments. The chronic care model has been used extensively to guide research on patients with T2DM, and although applying the model to HIV was novel, it was appropriate to guide the design of the HIV self-management intervention [21, 22]. This study was also influenced by Brown et al.’s (2015) model of diabetes self-management, which is based on the findings from a meta-analysis. In her model, social context, psychological factors (i.e., stress, depression, and knowledged), and behavioral factors (i.e., social support and self-management behaviors) predict intermediate-term outcomes, such as A1c level.

### Instruments

Data were collected twice: pre-test was at baseline and time 2 was within 2 weeks of completing the 12-week intervention. Descriptive data were gathered on participants’ age, gender, ethnicity/race, and length of diagnosis for both HIV and T2DM. Two questions assessed adherence to medication treatment on a visual analog scale: participants were asked to indicate the percentage of their adherence to treatment in the past 2 weeks, first for their HIV medication, then for their T2DM medication. Diabetes self-management activities were measured using the Summary of Diabetes Self-Care Activities scale (SDSCA) [20], an 11-item instrument that asks about the frequency in the last 7 days of completing various behaviors including T2DM glucose testing, exercise, and smoking.

Diabetes knowledge was measured using the Diabetes Knowledge Test (DKT) [21], with multiple choice items on two subscales: (a) a 14-item general test and (b) a 9-item insulin-use scale. The DKT had subpar reliability in the present study (Cronbach’s alpha = .39). HIV knowledge was measured using the HIV Knowledge Questionnaire (HIV-KQ-18) [23], with 18 true/false items that address risk, behavior change, and informed decisions. The internal consistency for the HIV-KQ with this sample was just under acceptable reliability (Cronbach’s alpha = .69).

We collected blood samples to assess diabetes control and HIV control. For diabetes control, A1c at baseline and at 12 weeks follow-up is used as an indicator for the previous 3 months of control, which is sufficient to show a change in A1c [24]. HIV control was assessed using viral load, which indicates the level of virus in the body, and CD4, which indicates immune system function. The goal is to have an undetectable viral load (< 20 copies/ml, per lab protocol; if the virus is suppressed, it greatly decreases transmission of the virus to others) and to have a CD4 level greater than 500 [25].

### Data analysis

Data were entered into SPSS 24 for Windows and checked for accuracy and missing data. We then assessed the instruments’ reliabilities and completed descriptive statistics to describe the sample.

## Results

### Focus group

Participants expressed an interest in learning more about how diabetes and HIV worked in the body, how to prepare healthy food and avoid sugar, how to manage depression and stress, and strategies to improve adherence to the medication regimen. Having examined findings from the focus group, we adapted the original diabetes intervention to include strategies to self-manage both HIV and T2DM, as well as components (such as games and other activities) that participants stated were important. We selected the location for the educational sessions and the time on the basis of participants’ feedback. The focus group participants said that they would be willing to attend hour-long sessions at the clinic in evenings over a 6-week period. The majority were unable to commit to a longer intervention.

### Adapted intervention

The intervention was adapted from Kim et al.’s [15] community-based self-management intervention for diabetes, which consisted of 12 h of instruction on diabetes self-management and six follow-up telephone calls. We tailored Kim et al.’s intervention for people with HIV by adding components that are key to self-management of both HIV and T2DM (e.g., diet, medications) and addressing topics specific to HIV self-management, including managing stigma and disclosure of status. We also streamlined intervention sessions to include topics that the focus group participants were most interested in. The original study was conducted at a cultural center regularly visited by participants with a history of retention into research studies. That site was used for many community participatory research projects [26, 27], whereas the present study took place at an urban HIV clinic. Barriers for participants in the present study to attend an intervention were greater than those for the general population [28, 29]. The revised intervention still adhered to the national standards of diabetes self-management education [30]. The result was a 12-week intervention that provided 6 h of educational sessions for groups of approximately six to eight participants followed by six follow-up weekly telephone calls. Participants received a cash incentive for every educational session they attended. The topics included pathophysiology of HIV and T2DM, medications, diet, depression, and relevant lab tests for HIV + T2DM. Each topic was introduced interactively using case studies, role-play, games, and group discussions (see Table 1). The intervention sessions concluded with an unfolding case study that contained information about all the topics covered. A registered nurse or licensed social worker led the groups and made six follow-up telephone calls to participants, in which they solicited participants’ questions about each of the intervention topics and re-explained or reinforced the intervention content. Telephone calls lasted about 10 min each. The interventionists were not members of the clinic staff nor the non-profit organization. They received training on HIV, diabetes, and self-management prior to starting the intervention and prior to each session by the primary investigator. An additional research member attended the educational sessions to take notes and ensure intervention fidelity.

**Table 1:** Topics for PLWH+DM educational intervention

Evidence-based intervention components	PLWH+DM tailored strategies
Understanding T2DM	Understanding the connection between DM and HIV. Comparing HIV and DM symptoms, complications, and medication side effects
Management principles of T2DM	Addressing understandings/misguided information about management principles of HIV
Diet	Integrating dietary recommendations with information that addresses common symptoms and side effects, including nausea and mouth sores. Stress low carbohydrates and increase in fruits and vegetables
Medications	HIV medications and the need for adherence to prevent resistance. DM medication adherence and blood glucose check when appropriate
Mental health/stress management	Acknowledging and appreciating stress associated with the development of HIV and accompanying illnesses Acknowledging level of stigma, mental health issues, and relationship of stress to managing HIV-T2DM
Mobilization of social support by nurse counselor	Enhancing individualized self-care skills including self-monitoring, adherence, and problem solving, by addressing barriers to care, HIV transmission, stigma, available resources

### Intervention results

The 25 PLWH+T2DM enrolled as participants in this study were predominantly male (65.4%), with a mean age of 56.6 (SD = 9); single or not currently in a relationship (84.6%); high school educated (69.2%); African American (57.7%); and taking oral medication for T2DM (65.4%). Approximately 20% identified as Latino (see Table 2). At baseline, fewer than half of the patients (46.7%) had an A1c level under 6.5%, and more than half had an undetectable HIV viral load (60%) (Table 3). Participants scored low on diabetes knowledge (mean score = 50%) and HIV knowledge (mean score = 62%), and they had low engagement in diabetes self-management behaviors (mean score = 40%). At baseline, self-management behaviors performed least often were to check one’s shoes (2.15, SD = 3.05), participate in an exercise (2.46, SD = 2.53), and check one’s blood sugar as often as was recommended by one’s providers (2.75, SD = 3.02). The most performed behavior in the previous 7 days was to check one’s feet. Inspection of feet and shoes is critical for patients with diabetes and is recommended daily to prevent ulcers and skin injury secondary to diabetic neuropathy [31]. Foot ulcers can be costly and in the most serious complication can result in amputation [32].

**Table 2:** Demographic characteristics of participants (*N* = 25)

Characteristic	Number	Percent
Gender		
Male	17	68
Female	7	24
Other	1	4
Education		
Junior high	2	8
High school	18	72
Associates/some college	1	4
College degree	4	16
Relationship status		
Married or committed relationship	3	12
Divorced	3	12
Widowed	2	8
Single	17	68
Race/ethnicity		
White	8	30.8
African American	15	58.7
Latino	5	19.2
Other	2	7.7
Medication regimen		
Insulin	11	42.6
Oral medication	17	65.4
Diet/exercise only	7	26.9
A1c below 6.5	8	53.3
Undetectable viral load	9	60.0

**Table 3:** Pre- and posttest results

	Pretest		Posttest		
	Mean	SD	Mean	SD	*p*
Diabetes knowledge (DKT)	11.4	4.2	10.4	3.5	.505
HIV knowledge (HIV-KQ-18)	11.3	5.1	12.5	3.7	.491
Diabetes self-management activities (SDSCA)	30.8	12.7	41.25	7.8	.053

*DKT* Diabetes Knowledge Test, *HIV-KQ-18* HIV Knowledge Questionnaire, *SDSCA* Summary of Diabetes Self-Care Activities

The first round of the intervention was offered to a cohort of eight participants in six weekly 1-h sessions held in a private conference room at the clinic one evening per week. A light meal was provided. These eight participants attended 48% of the sessions. Participants indicated that transportation and competing demands on their time made the six weekly sessions inconvenient. Therefore, for the second and third cohorts of 8–9 participants, the intervention was offered in two 3-h meetings held in the evening at the clinic 2 weeks apart. Participants completed 2.4 of the 6 h offered to cohorts 2 and 3 and an average of 40% of the sessions. Overall, participants completed an average of 45% hours of the 6-h group intervention and participated in an average of three of the six available support telephone calls from the registered nurse or social worker.

Most participants either completed all or none of the sessions in either format. We compared baseline characteristics of participants who completed all 6 h of the intervention (*n* = 9) with baseline characteristics of those who completed fewer than 6 h (*n* = 14). There were no significant differences in age, knowledge of either T2DM or HIV, social support, diabetes self-management behaviors, or depression between high-attendance and low-attendance participants.

## Discussion

The primary objectives of this pilot study were twofold. First was to adapt an existing, evidence-based diabetes self-management intervention for PLWH+T2DM. The focus group helped select preferences and topics. Although most self-management tools for T2DM are similar to those for HIV [16], some topics would not be included in a self-management intervention for just one condition. The focus group discussed a need for managing symptoms that could be attributed to either condition, and we believe that a comorbidity self-management intervention can improve health outcomes.

The second purpose was to evaluate the feasibility of administering the intervention to a sample of PLWH+T2DM from a low-income, predominantly minority, vulnerable population. The pilot study was able to recruit participants that reflected the area’s population in a few months. The baseline data highlight the need for an intervention to improve these participants’ self-management skills. Their knowledge of T2DM and HIV was low; they were able to answer about half of the questions about either condition correctly. Furthermore, the participants performed only 40% of recommended diabetes self-management activities, which is lower than reported in seven other studies [33]. For example, in Toobert et al.’s [33] review of seven studies in which the SDSCA was used, the mean blood glucose testing subscale score was 69.0, but in the present study the mean was 54.9, and participants reported completion of more self-management behaviors after the intervention, especially checking insides of shoes for rough areas. Baseline knowledge, diabetes and HIV control, and self-management behaviors point to a clear need for a self-management intervention for PLWH+T2DM.

Secondary objectives were to create acceptable procedures for implementation. Although we were able to recruit PLWH+T2DM and implement the intervention, attendance was low. The barrier named most often by the participants was transportation to the intervention site, even though the intervention was held at a clinic where many of the participants were patients, located on a major highway, and accessible by three bus lines. Travel to this location could be burdensome for those who do not live close to the city center.

To increase attendance, prior to each meeting, we reminded participants about the intervention and confirmed their attendance. We provided cash incentives for completing the intervention ($25 per session) and data collections ($30 per study visit), which participants could use for transportation or other preferences. Future studies should consider providing patient transportation, such as car shares or taxis. Another option would be to provide the intervention in a given location at a time when the participants would already be waiting. For example, the research team recruited many of the participants from the local food bank for PLWH, which had long waiting times. The intervention’s educational instruction might have been provided while participants were waiting for the food bank to open.

After the first cohort, we modified the delivery format to accommodate participants’ schedules by offering two 3-h sessions instead of the original six 1-h sessions; however, attendance did not improve with the revised format. A hybridized classroom, with some of the educational instruction self-paced at home and some of the session in person, might have made it possible for participants to receive more of the educational material. The use of technology to provide instruction might also address the barrier to attending educational sessions in person.

We planned to obtain pre/post blood samples from the participants, but without success. The present study followed recommended blood collection procedures used in a previous study that had a 100% participation rate, but the protocol was not successful with the present participants. Fifteen of the 25 participants participated in the baseline fasting blood tests at a commercial lab, but only one completed the blood draw for the follow-up data collection. Therefore, we cannot report the effects of the intervention on HIV or diabetes control. It is not known whether participants had the capacity or the inclination to complete that step of participation. Rather than have participants take the extra step of going to the laboratory, data collection rates might be improved by having the research team draw the blood samples upon completion of the study surveys or by awarding separate monetary incentives specifically for blood draws. Future studies will consider this barrier when creating blood draw protocols.

To overcome the low attendance and low laboratory draw participation, future studies may also need to address known barriers to access such as housing instability, low health literacy, and reduced cognitive function [34–36]. Although these social determinants might not seem to affect participation directly, such challenges might be distracting or affect research participation in other ways. The intervention protocol may need modifications to improve participants’ attendance so that they receive the intervention’s full dose. Participation may improve if the intervention is scheduled with events that participants are already attending, such as clinic appointments or community events.

The other secondary objective was to investigate the instrument’s usefulness in the dual diagnosis population. Scores on diabetes knowledge did not improve posttest. The Diabetes Knowledge Test itself may have hindered the findings and may be a poor fit for this sample population. First, the Cronbach alpha was marginally low, which can indicate an unreliable scale. Secondly, The DKT asks 9 questions about insulin, the study sample did not use insulin to manage their diabetes and the intervention had no education on insulin. Fitzgerald et al. [37] reported a Cronbach’s alpha of ≤ .70; in the present study, the DKT achieved a Cronbach’s alpha of .69. Compared to Fitzgerald et al.’s study, more of those participants were White and female, though the percentage of people not using insulin was about the same. Finally, the percentage of correct questions was higher for all three groups in Fitzgerald et al.’s sample than in the present study (88.57%, 68.27%, and 66.54% vs 49.6%). The DKT was revised; the subscales are now scored separately and some of the questions were changed for clarity [38]. This newer version may be a better fit for this population.

## Limitations

This was a pilot study, with limitations that will need to be addressed in a larger project. Five participants were members of the focus group as well as the intervention group, so they may have been more interested in the study’s feasibility than were other participants. Future studies could exclude focus group participants. Conducting a second focus group in the population outside the clinic might provide more information about the location, timing, and modality of educational sessions. This information could potentially improve attendance and retention, because some of the participants did not prefer the clinic’s location; indeed, some reported negative associations with the clinic. The low retention may have indicated participants’ level of interest in a self-management in-person intervention; the instruments used to measure knowledge may have been inappropriate for this population.

## Next steps

The next step will be to revise the intervention with the lessons learned from this pilot. We will make the intervention multi-modal, using a combination of in-person, at-home, and teleconferencing procedures that should allow for flexibility in attendance. The original intervention had 6 more hours of educational instruction. A multi-modal intervention will allow us to increase hours of instruction without increasing the time commitment for an in-person intervention. Additionally, we will need to create and test a dual-condition knowledge test for diabetes and HIV that includes knowledge specifically tailored for this combination.

## Conclusions

Interventions to improve self-management of diabetes in PLWH are needed in this community, given low levels of knowledge and self-management behaviors that need to be addressed to improve health outcomes. The intervention’s format and procedures must be revised to retain participants and to meet their needs. Barriers to attendance and obtaining blood specimens will be addressed in subsequent research.

## Data Availability

All data will be available upon request.
